# Supplementary material for: Structure-Activity Relationship Investigations of Novel Constrained Chimeric Peptidomimetics of SOCS3 Protein Targeting JAK2
Source: Pharmaceuticals (Basel). 2022 Apr 9;15(4):458. doi: 10.3390/ph15040458 (PMC9031227; doi:10.3390/ph15040458)
Supplement: Supplementary file 1 [file pharmaceuticals-15-00458-s001.zip › pharmaceuticals-1664741-supplementary.pdf]

SUPPLEMENTARY MATERIALS

Structure-Activity Relationship investigations of novel constrained chimeric peptidomimetics of SOCS3 protein targeting JAK2

Sara La Manna[1], Marilisa Leone[2], Flavia Anna Mercurio[2], Daniele Florio[1] and Daniela Marasco[1,*]

[1] Department of Pharmacy, CIRPEB: Research Center on Bioactive Peptides- University of Naples "Federico II", 80131, Naples, Italy; sara.lamanna@unina.it, daniele.florio@unina.it, daniela.marasco@unina.it
[2] Institute of Biostructures and Bioimaging (CNR), Naples, Italy; marilisa.leone@cnr.it, flaviaanna.mercurio@cnr.it

Table of contents:

Figure S1. 1D [1H] and 2D [1H-1H] NOESY 300 spectra of KIRCONG *i/i+5* in H_2O/TFE 85/15 v/v and in H_2O/TFE 60/40 v/v.

Figure S2. 2D [1H-1H] TOCSY and NOESY 300 spectra of KIRCONG *i/i+7* in H_2O/TFE 60/40 v/v.

Figure S3. NMR structures of KIRCONG *i/i+7* in H_2O/TFE (60/40 v/v) and KIRCONG chim in H_2O/TFE (60/40, v/v).

Figure S4. Capillary shape and capillary scan of designed analogues.

Figure S5. Binding isotherms for MST signals versus linear KIRCONG *i/i+5* concentrations.

Table S1. Deconvolution of CD spectra.

Table S2. ^{1}H chemical shifts of KIRCONG *i/i+5* in H_2O/TFE (60/40, v/v).

Table S3. ^{1}H chemical shifts of KIRCONG *i/i+5* in H_2O/TFE (85/15, v/v).

Table S4. ^{1}H chemical shifts of KIRCONG *i/i+7* in H_2O/TFE (60/40, v/v).

Table S5. Structure statistics of KIRCONG *i/i+7* NMR conformers in H_2O/TFE (60/40, v/v).

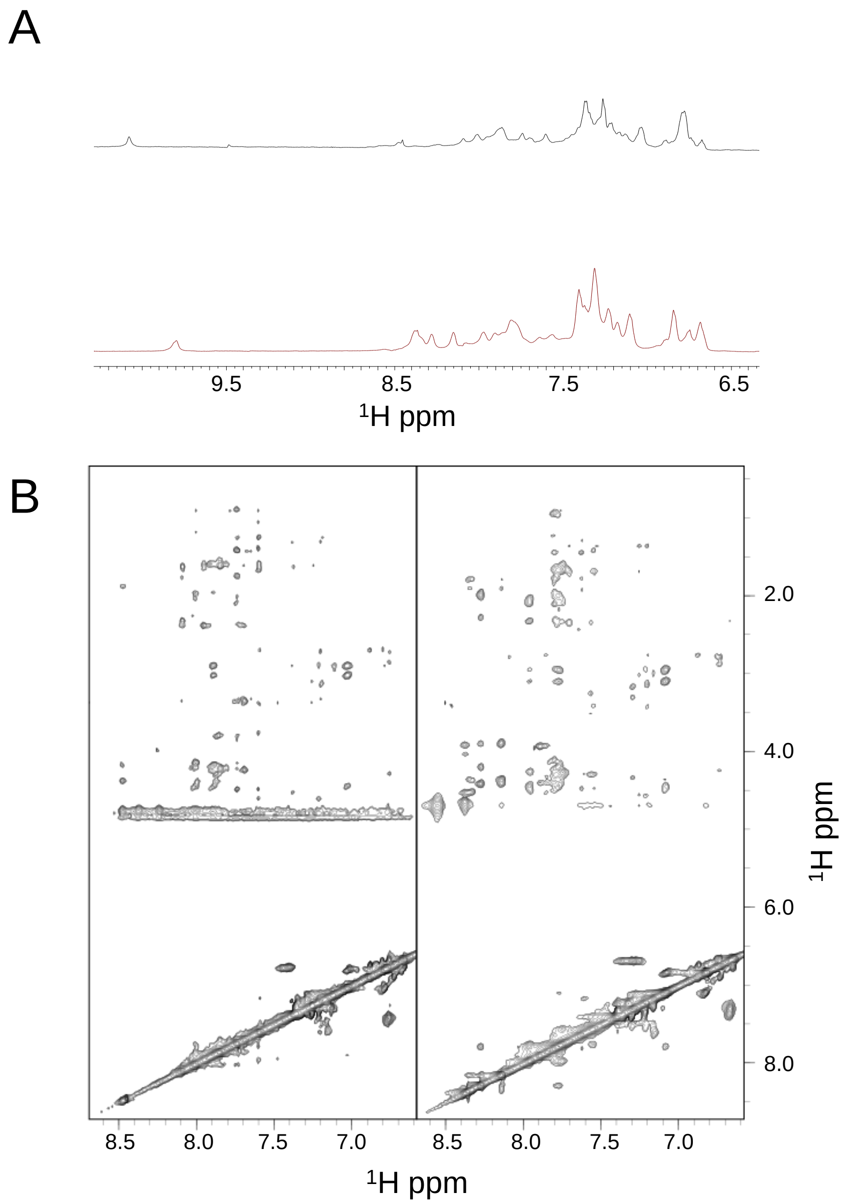

Figure S1. (**A**) Comparison of 1D [^{1}H] spectra of KIRCONG *i/i+5* in H_2O/TFE 85/15 v/v (black) and KIRCONG *i/i+5* in H_2O/TFE (60/40 v/v) (red). Spectral regions containing peaks from H_N and aromatic protons are shown. (**B**) 2D [^{1}H-^{1}H] NOESY 300 spectra of KIRCONG *i/i+5* in H_2O/TFE 85/15 v/v (left panel) and KIRCONG *i/i+5* in H_2O/TFE 60/40 v/v (right). The figure shows spectral regions containing correlations involving H_N, aromatic and aliphatic protons.

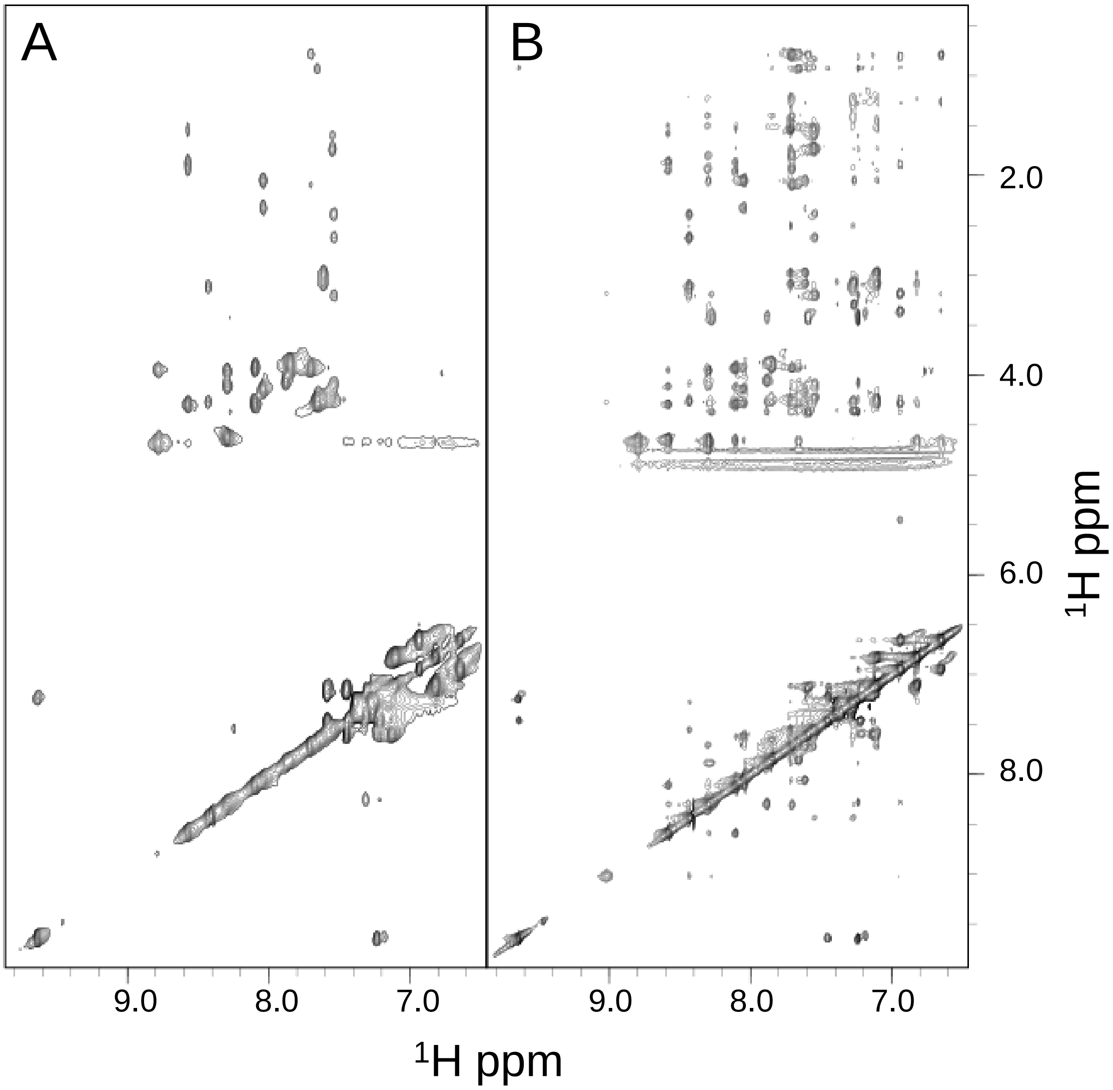

Figure S2. 2D [^{1}H-^{1}H] TOCSY (**A**) and NOESY 300 (**B**) spectra of KIRCONG *i*/*i+7* in H_2O/TFE 60/40 v/v. The figure shows spectral regions containing correlations involving H_N, aromatic and aliphatic protons.

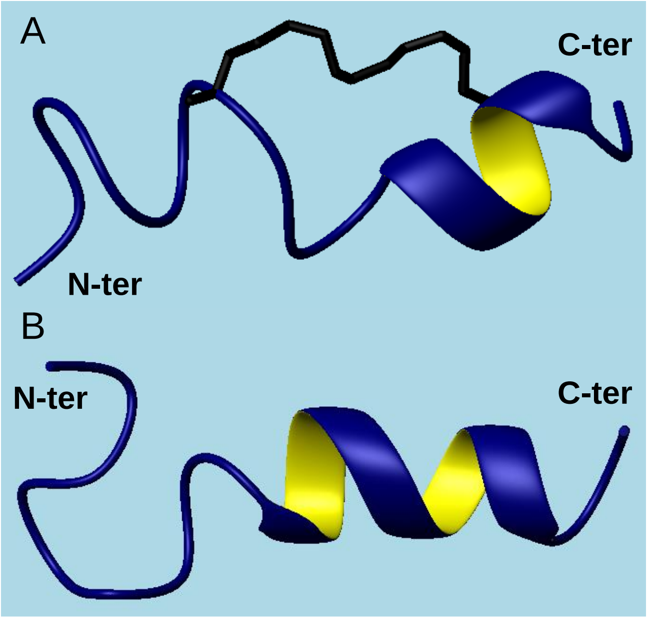

Figure S3. NMR structures (conformers n. 1) of KIRCONG *i*/*i*+7 in H_2O/TFE (60/40, v/v) (**A**), and KIRCONG chim in H_2O/TFE (60/40, v/v) (**B**).

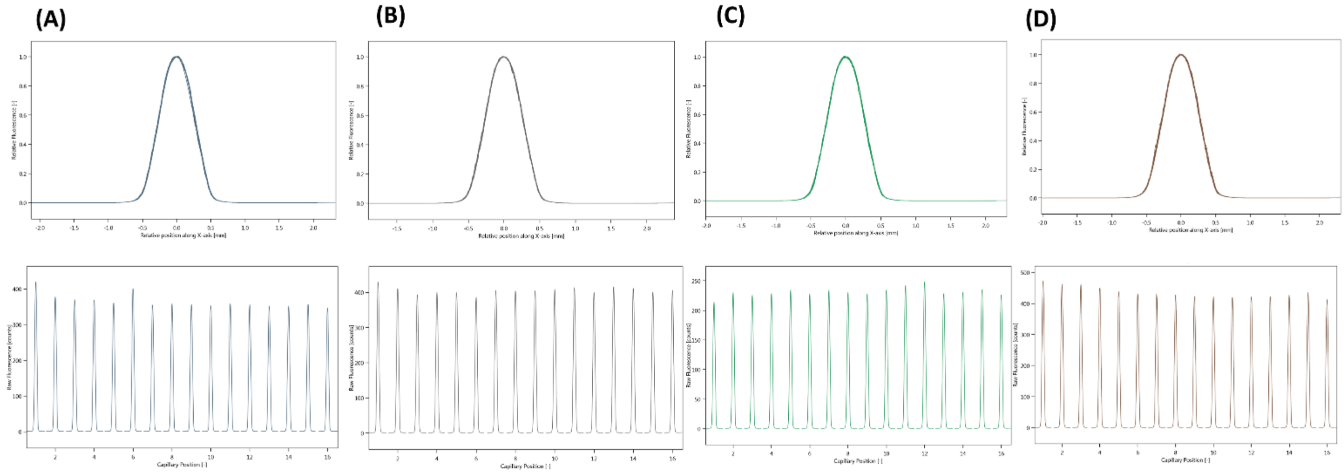

Figure S4. Upper: capillary shape and lower: capillary scan of: (**A**) KIRCONG *amide*, (**B**) KIRCONG *disulfide*, (**C**) KIRCONG *i*/*i*+5 and (**D**) KIRCONG *i*/*i*+7.

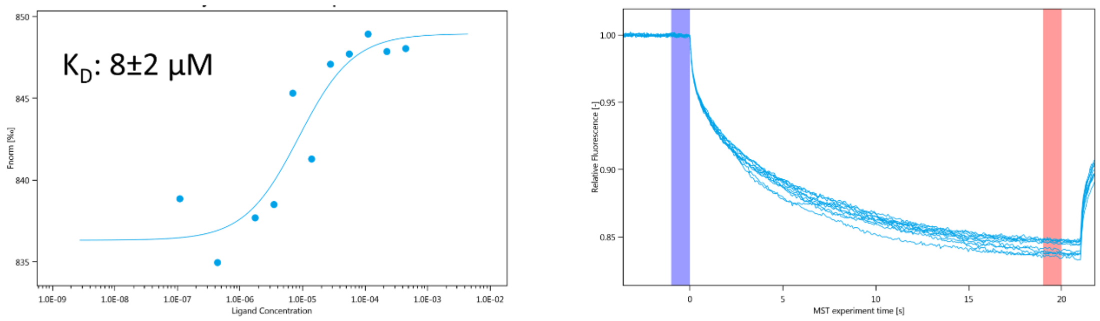

Figure S5. Binding isotherms for MST signals versus linear KIRCONG *i*/*i*+*5* concentrations.

Table S1. Deconvolution of CD spectra.

	%TFE	HELIX	BETA	TURN	OTHERS
**KIRCONG *amide***	15	0	42.0	12.7	45.4
	25	0	41.5	13.6	44.9
	45	1.3	39.7	14.9	44.1
	65	8.3	31.8	14.5	45.4
**KIRCONG *disulfide***	15	0.6	43.8	12.9	42.7
	25	0	42.6	13.6	43.8
	45	9.8	35.0	13.4	41.8
	65	8.3	34.7	13.4	43.6
**KIRCONG *i*/*i*+5**	15	8.2	37.4	13.6	40.8
	25	6.1	37.5	13.9	42.4
	45	22.6	21.0	13.3	43.1
	65	41.5	16.8	9.9	31.7
**KIRCONG i/*i*+*7***	15	3.8	43.7	13.6	39
	25	12.1	31.8	13.5	42.7
	45	24.4	23.5	13.4	38.7

Table S2. ^{1}H chemical shifts of KIRCONG *i*/*i*+5 in H_2O/TFE (60/40, v/v), pH 4.52, and T=25°. In red tentative assignments are reported. The β-Alanine and (S)-N- 2-(4'-pentenyl) alanine are indicated as "BAL" and "X", respectively. N.D. stands for not determined.

Residue	HN	Hα	Hβ	Hγ	Others
**1F**	N.D.	4,34	3,17-3,30		δ 7,30<br>ε 7,40
**2S**	8,55	4,64	3,90-3,94		
**3S**	8,38	4,52	3,92-4,02		
**4K**	8,34	4,36	1,79-1,91	1,50	δ 1,72<br>ε 3,01
**5S**	8,15	4,42	3,89-3,91		
**6E**	8,28	4,21	1,96-2,01	2,29	
**7Y**	7,78	4,50	2,95-3,10		δ 7,09<br>ε 6,83
**8Q**	7,97	4,26	2,06-2,11	2,34	ε 6,68-7,31
**9L**	7,75	4,31	1,59-1,66	1,65	0,87-0,92
**10BAL**	7,57	2,35	3,28-3,51		
**11X**	7,70		βCH3 1,36<br>1,70-1,77	1,36	δ 1,69–1,78<br>Hε 5,26
**12F**	7,86	4,56	2,76-2,82		δ 7,22<br>ε 7,26
**13Y**	8,10	4,23	2,80-2,88		δ 6,74<br>ε 6,90
**14W**	7,53	4,55	2,95-3,03		Hε1 9,80<br>Hδ1 7,21<br>Hη2 7,29<br>Hε3 7,61<br>Hζ3 7,17<br>Hζ2 7,31
**15S**	7,85	4,47	3,94		
**16X**	7,63		βCH3 1,46<br>1,78-1,94	1,33	δ 1,77–1,92<br>Hε 5,40
**17V**	7,80	4,10	2,09	0,97-1,01	
**18T**	7,83	4,36	4,38	1,24	
**19G**	7,90	3,93			

Table S3. ^{1}H chemical shifts of KIRCONG *i/i+5* in H_2O/TFE (85/15, v/v), pH 6.84, and T=25°. In red tentative assignments are reported. "BAL" and "X" are used for β-Alanine and (S)-N- 2-(4'-pentenyl) alanine, respectively. N.D. stands for not determined.

Residue	HN	Hα	Hβ	Hγ	Others
**1F**	N.D.	4,34	3,17-3,30		δ 7,13<br>ε 7,30
**2S**	N.D.	4,74	3,97		
**3S**	N.D.	4,56	3,84		
**4K**	8,37	4,32	1,75-1,86	1,41	δ 1,64<br>ε 2,96
**5S**	8,24	4,37	3,83-3,87		
**6E**	8,47	4,16	1,88-1,93	2,14	
**7Y**	7,89	4,44	2,89-3,02		δ 7,03<br>ε 6,79
**8Q**	8,00	4,21	1,96-2,02	2,27	ε 6,77-7,42
**9L**	7,85	4,24	1,54-1,61	1,58	0,83-0,89
**10BAL**	7,69	2,38	3,35		
**11X**	7,60		βCH3 1,36<br>1,73	1,66	δ 1,651,78<br>Hε 5,27
**12F**	7,86	4,60	2,69-2,72		δ 7,22<br>ε 7,34
**13Y**	8,10	4,26	2,72-2,85		δ 6,76<br>ε 6,89
**14W**	7,59	4,59	2,70-2,91		Hε1 10,08<br>Hδ1 7,21<br>Hη2 7,44<br>Hε3 7,59<br>Hζ3 7,17<br>Hζ2 7,41
**15S**	7,85	4,47	3,94		
**16X**	7,74		βCH3 1,40<br>1,73-1,76	1,07	δ 1,68–1,78<br>Hε 5,34
**17V**	7,75	4,13	2,11	0,89-0,91	
**18T**	8,00	4,37	4,31	1,20	
**19G**	7,86	3,79			

Table S4. [1]H chemical shifts of KIRCONG *i*/*i*+7 in H_2O/TFE (60/40, v/v), pH 4.65, and T=25°C. "BAL", "X" and "Z" indicate β-Alanine, (S)-N-2-(4'-pentenyl) alanine, and (R)-N- 2-(7'-octenyl) alanine, respectively. N.D. stands for not determined.

Residue	HN	Hα	Hβ	Hγ	Others
**1F**	N.D.	4,35	3,07-3,31		δ 7,27<br>ε 7,39
**2S**	8,78	4,68	3,95-3,97		
**3S**	8,29	4,63	3,96-4,11		
**4K**	8,57	4,28	1,88-1,96	1,51-1,58	δ 1,72<br>ε 3,00
**5S**	8,10	4,29	3,91-3,95		
**6E**	8,04	4,13	2,03-2,06	2,32-2,35	
**7Y**	7,72	4,23	2,98-3,08		δ 7,40<br>ε 6,83
**8Z**	8,00		βCH3 1,54<br>1,74	1,22-1,45	δ 1.47–2.51<br>ε 1,93<br>η 1.28<br>ζ 1.89<br>Hζ1 5.45
**9L**	7,56	4,24	1,59-1,75	1,71	0,86-0,94
**10BAL**	7,55	2,39-2,62	3,19-4,10		
**11F**	8,44	4,27	3,12		δ 7,27<br>ε 7,38
**12Y**	N.D.	4,28	3,18-3,36		δ 6,94<br>ε 6,66
**13W**	8,27	4,36	3,41-3,46		Hε1 9,63<br>Hδ1 7,25<br>Hη2 7,21<br>Hε3 7,58<br>Hζ3 7,13<br>Hζ2 7,45
**14S**	7,88	4,07	3,87		
**15X**	8,31		βCH3 1,41<br>1,80-1,93	1,24-1,51	δ 1,87–2,10<br>Hε 5,37
**16V**	7,70	3,93	2,09	0,79-0,82	
**17T**	7,66	4,27	4,19	0,94	
**18G**	7,85	3,89			

Table S5. Structure statistics of KIRCONG *i/i+7* NMR conformers in H_2O/TFE (60/40, v/v).

**Residual target function, Å²**	0.27±0.05
**Residual NOE violations**	1
Number ≥ 0.1 Å*	0
**Residual angle violations**	0
**Atomic pairwise RMSD, Å**	
Backbone atoms (residues 3-17)	0.53±0.15
Heavy atoms (residues 3-17)	1.03±0.16
**Procheck analysis (all residues)#**	
Residues in core regions	49.7%
Residues in allowed regions	35.3%
Residues in generous regions	14.1%
Residues in disallowed regions	0.9%

* CYANA [1] average violations
#PROCHECK_NMR [2] statistics

References

1. Herrmann, T.; Guntert, P.; Wuthrich, K. Protein NMR structure determination with automated NOE assignment using the new software CANDID and the torsion angle dynamics algorithm DYANA. *J Mol Biol* **2002**, *319*, 209-227.
2. Laskowski, R.A.; Rullmannn, J.A.; MacArthur, M.W.; Kaptein, R.; Thornton, J.M. AQUA and PROCHECK-NMR: programs for checking the quality of protein structures solved by NMR. *J Biomol NMR* **1996**, *8*, 477-486.
